# Predictors of next-generation sequencing panel selection using a shared decision-making approach

**DOI:** 10.1038/s41525-018-0050-y

**Published:** 2018-04-27

**Authors:** Eliza Courtney, Shao-Tzu Li, Tarryn Shaw, Yanni Chen, John Carson Allen, Joanne Ngeow

**Affiliations:** 1 Cancer Genetics Service, Division of Medical Oncology, National Cancer Centre Singapore, Singapore, 169610 Singapore; 2 Centre for Quantitative Medicine, Duke-NUS Medical School, Singapore, 169857 Singapore; 3 Lee Kong Chian School of Medicine, Nanyang Technological University, Singapore, 308232 Singapore; 4 Oncology Academic Clinical Program, Duke-NUS Medical School, Singapore, 169857 Singapore; 5 Institute of Molecular and Cell Biology, Agency for Science Technology and Research (A*Star), Singapore, 138673 Singapore

## Abstract

The introduction of next-generation sequencing panels has transformed the approach for genetic testing in cancer patients, however, established guidelines for their use are lacking. A shared decision-making approach has been adopted by our service, where patients play an active role in panel selection and we sought to identify factors associated with panel selection and report testing outcomes. Demographic and clinical data were gathered for female breast and/or ovarian cancer patients aged 21 and over who underwent panel testing. Panel type was classified as ‘breast cancer panel’ (BCP) or ‘multi-cancer panel’ (MCP). Stepwise multiple logistic regression analysis was used to identify clinical factors most predictive of panel selection. Of the 265 included subjects, the vast majority selected a broader MCP (81.5%). Subjects who chose MCPs were significantly more likely to be ≥50 years of age (49 vs. 31%; *p* < 0.05), Chinese (76 vs. 47%; *p* < 0.001) and have a personal history of ovarian cancer (41 vs. 8%; *p* < 0.001) with the latter two identified as the best predictors of panel selection. Family history of cancer was not significantly associated with panel selection. There were no statistically significant differences in result outcomes between the two groups. In summary, our findings demonstrate that the majority of patients have a preference for interrogating a larger number of genes beyond those with established testing guidelines, despite the additional likelihood of uncertainty. Individual factors, including cancer history and ethnicity, are the best predictors of panel selection.

## Genetic testing: Understanding cancer patient preferences

Thanks to next-generation sequencing (NGS) it is possible to test multiple genes for cancer susceptibility quickly and cheaply. At the National Cancer Centre Singapore, shared decision-making is used to actively encourage the involvement of patients in the selection of either a ‘breast cancer panel’ (BCP) or a broader ‘multi-cancer panel’ (MCP) test. Joanne Ngeow at the Centre’s Cancer Genetics Service and colleagues examined the choices made by 265 patients with breast and/or ovarian cancer following detailed pre-test counselling by the genetics team. The majority of patients selected the MCP, potentially highlighting a willingness to accept the additional uncertainty that comes with broader NGS panels. Interestingly, a personal history of ovarian cancer and Chinese ethnicity, but not a family history of cancer, were significantly associated with panel selection.

## Introduction

Over the past few decades, our understanding regarding the hereditary nature of cancer has evolved considerably, and over 200 hereditary cancer syndromes have now been described.^1,2^ With the advent of next-generation sequencing (NGS), cancer genetic testing has transformed from phenotype-directed single-gene testing to comprehensive panel testing, where multiple genes are interrogated simultaneously. The immediate benefits are manifold, including dramatic reductions in the duration and cost of testing, as well as the generation of significant amounts of information regarding cancer susceptibility from a single DNA sample.^3–5^ The potential exists for more patients to be given a genetic diagnosis sooner and thus be spared testing fatigue—the ‘diagnostic odyssey’.^4,6–8^

While there is great optimism for NGS panel testing, there are still a number of limitations to be considered. In addition to high-penetrance genes, NGS panels typically include a mixture of moderate- and low-penetrance genes, many of which are without well-established cancer risk estimates and medical management guidelines.^3,5^ As opposed to phenotype-directed single-gene testing, NGS panel testing creates opportunity for unexpected findings, where a pathogenic variant is identified in a gene that is unrelated to the patient’s personal or family history.^9,10^ It is unclear whether risk management protocols should be altered in these cases. Additionally, the likelihood of detecting variants of uncertain significance (VUS), which may or may not impact the gene function, rises significantly with NGS panel testing.^4,7,11,12^ This is both due to the number of genes tested and due to our limited understanding of what constitutes normal genetic variation, especially for newly discovered moderate- and low-penetrance genes. Furthermore, there is also an added complexity that comes with testing multiple genes simultaneously involving the potential for pathogenic variants in more than one gene to be identified in a single patient.^4,13^ The possible gene–gene interactions and their influence on overall cancer risk is still a question that remains unanswered.

Given the abundance of uncertainty that NGS panel testing can generate, there has been much debate regarding its application into routine clinical care. There is still no international consensus for NGS panel use, although a number of professional bodies have published recommendations for the use of NGS technologies.^3,14–17^ In its 2015 position statement, the American Society of Clinical Oncology stated that, while it was sufficient to offer the testing of genes with proven clinical utility as guided by the patient’s personal and/or family history, broader NGS panels, including genes not clinically indicated or with little evidence of clinical utility, should only be offered by clinicians with appropriate expertise in cancer-risk assessment.^3^ Informed consent remains fundamental to the pre-test counseling process, and various models for delivering the information have been suggested.^3,5,18^ Panel selection is often determined by service providers or health care insurers.

Shared decision-making (SDM) provides one possible strategy for NGS panel selection. In SDM, decisions regarding medical interventions are made collaboratively between clinician and patient by balancing the potential benefits and risks with the patient’s own values and preferences.^19^ The use of SDM in genetic counseling is not a new concept, and was first postulated as a useful model to compliment non-directive counseling almost two decades ago.^20^ Published studies on the application of SDM in cancer genetics services have mainly focused on the use of decision aids regarding the choice to pursue genetic testing^21^ or the subsequent risk-management options for those at increased risk of cancer.^22^ To the best of our knowledge, SDM has not been investigated when applied to the selection of NGS panels.

The effectiveness of SDM in clinical care can be undermined by a number of factors and uncertainty has been hypothesized as one example.^23,24^ The way in which clinicians and patients respond to uncertainty has the potential to alter the way therapeutic relationships are formed, and how information is shared. Indeed, a significant association has been demonstrated between patients’ decision satisfaction (a common measure in SDM research) and clinicians’ anxiety resulting from uncertainty.^25^ Additionally, a negative correlation between the amount of uncertainty communicated by clinicians and decision satisfaction amongst cancer patients has been reported.^26^ Importantly, however, the same study found that decision satisfaction increased in patients with greater involvement in the decision-making process, suggesting that SDM may have a role in alleviating some of the potential burden posed by uncertainty.

The Cancer Genetics Service (CGS) at the National Cancer Centre Singapore (NCCS) has implemented the use of NGS panel testing into routine clinical care. The CGS has adopted a SDM approach where patients play an active role in the selection between a NGS panel, based on clinical indication and utility, and a broader panel covering a larger number of genes. The testing options, potential benefits and harms are presented to the patient and explored in the context of the patient’s own values and preferences. The present descriptive study sought to present the overall patterns of NGS panel selection in relation to demographic and clinical variables using a SDM approach. The results will provide the necessary foundations on which to build further research; to explore the various impacts and outcomes when involving the patient in the decision-making process. As panel selection is a challenging aspect of the delivery of cancer genetics services, this is a pertinent area for investigation. The results will provide clinicians and policy makers with insights into the potential factors influencing patients in their decision-making and presents real-life data for the application of SDM to NGS panel selection. This will contribute to the understanding of patient preferences and thus may have implications for the future development of NGS panel guidelines.

## Results

### Subject characteristics

Subject characteristics are presented in Table 1. The majority of the subjects were under age 50 (54.0%), Chinese (70.9%), and parous (71.3%). The ethnic distribution is representative of the Singaporean population.^27^ The majority of subjects had a personal history of breast cancer and 20 a personal history of both breast and ovarian cancer (10.4% of breast cancer and 21.7% of ovarian cancer patients). Most had a family history of cancer (76.6%), with 157 (77.3%) involving cancer types other than breast and ovarian. Of the 47 subjects with a personal history of breast cancer who selected a breast cancer panel (BCP), 16 (34.0%) opted for testing with a fast turnaround time. The average number of appointments required to decide on testing was 1.5: 152 (57.4%) subjects proceeded with testing at their first appointment, 101 (38.1%) at their second, and 12 (4.5%) required three or more appointments.

**Table 1:** Demographics and clinical characteristics by NGS panel choice, n (%)

Variable	Category	Total (*N* = 265)	BCP (*N* = 49)	MCP (*N* = 216)	*p* value^a^
Demographics					
Age					
	<50	145 (54.7)	34 (69.4)	111 (51.4)	**0.0057**
	≥50	120 (45.3)	15 (30.6)	105 (48.6)	
Ethnicity					
	Chinese	188 (70.9)	23 (46.9)	165 (76.4)	**0.0003**
	Malay	24 (9.1)	6 (12.2)	18 (8.3)	
	Indian	13 (4.9)	3 (6.1)	10 (4.6)	
	Others	40 (15.1)	17 (34.7)	23 (10.6)	
Parous					
	Yes	189 (71.3)	37 (75.5)	152 (70.4)	0.4726
	No	76 (28.7)	12 (24.5)	64 (29.6)	
Personal history of cancer					
Breast cancer					
	Yes	193 (72.8)	47 (95.9)	146 (67.6)	**0.0009**
	No	72 (27.2)	2 (4.1)	70 (32.4)	
Ovarian cancer					
	Yes	92 (34.7)	4 (8.2)	88 (40.7)	**0.0002**
	No	173 (65.3)	45 (91.8)	128 (59.3)	
Family history of cancer in relatives^b^					
Any cancer type					
	Present	203 (76.6)	36 (73.5)	167 (77.3)	0.8350
	Absent	62 (23.4)	13 (26.5)	49 (22.7)	
Breast cancer only					
	Present	33 (12.5)	6 (12.2)	27 (12.5)	0.9508
	Absent	232 (87.5)	43 (87.8)	189 (87.5)	
Ovarian cancer only					
	Present	7 (2.6)	1 (2.0)	6 (2.8)	0.9985
	Absent	258 (97.4)	48 (98.0)	210 (97.2)	
Breast and/or ovarian cancer only					
	Present	46 (17.4)	8 (16.3)	38 (17.6)	0.9013
	Absent	219 (82.6)	41 (83.7)	178 (82.4)	
Colorectal and/or endometrial cancer					
	Present	54 (20.4)	8 (16.3)	46 (21.3)	0.8903
	Absent	211 (79.6)	41 (83.7)	170 (78.7)	

*BCP* breast cancer panel, *MCP* multi-cancer panel

^a^Fisher’s exact test

^b^Family history in first-, second-, and/or third-degree relatives

The bold values correspond to *p* values that are significant (i.e. < 0.05)

### Factors associated with NGS panel selection

The associations between NGS panel selection and demographic and clinical factors, investigated using Fisher’s exact test (FET), are presented in Table 1. Of the 265 subjects, 216 (81.5%) selected a multi-cancer panel (MCP). Subjects who selected MCPs vs. BCPs were more likely to be Chinese (76.4 vs. 46.9%; FET, *p* = 0.0003) and over 50 years of age (48.6 vs. 30.6%; FET, *p* = 0.0057). There was no significant association between NGS panel selection and parity status. Subjects with a personal history of ovarian cancer were more likely to select a MCP (40.7 vs. 8.2%; FET, *p* *=* 0.0002), while those with personal history of breast cancer were more likely to select a BCP (95.9 vs. 67.6%; FET, *p* *=* 0.0009).

Family history of cancer was not associated with NGS panel selection (FET, *p* *=* 0.8350). Even for subjects with a family history of breast, ovarian, and breast and/or ovarian cancer, FET showed no significant differences in proportions of patients selecting a BCP vs a MCP (12.2 vs. 12.5%, *p* *=* 0.9508; 2.0 vs. 2.8%, *p* *=* 0.9985; and 16.3 vs. 17.6%, *p* = 0.9013, respectively). Additionally, subjects selecting a MCP were marginally more likely to have a family history of colorectal cancer and/or endometrial cancer, although this association was not significant (21.3% vs. 16.3%; FET, *p* *=* 0.8903).

Stepwise multiple logistic regression identified ethnicity and personal history of ovarian cancer from among the demographic and clinical factors presented in Table 1 as the best independent predictors of NGS panel selection (Wald chi-square test: race, *χ*2 = 18.5, df = 3, *p* *=* 0.0003; personal history of ovarian cancer, *χ*2 = 14.9, df = 1, *p* = 0.0001). Area under the receiver operating characteristic (ROC) curve (95% CI) was AUC = 0.77 (0.70, 0.84).

### Spectrum of result outcomes

**Fig. 1:**
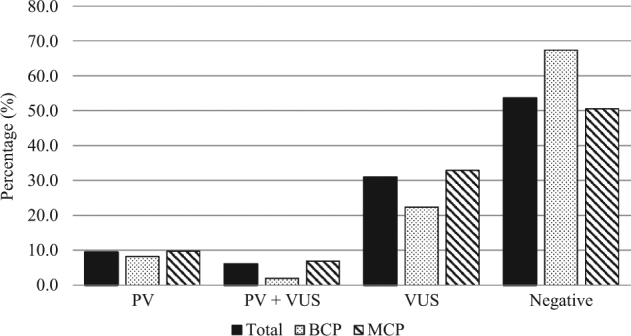
Proportions of result outcomes among the total cohort, and by NGS panel group. BCP breast cancer panel, MCP multi-cancer panel, PV pathogenic variant detected, PV + VUS pathogenic variant and variant of uncertain significance detected, VUS variant of uncertain significance detected; Negative, no variants (PV or VUS) detected

Overall, pathogenic variants were detected in 25 (9.4%) patients, pathogenic variants plus VUS in 16 (6.0%), and one or more VUS in 82 (30.9%); 142 (53.6%) subjects were negative with no pathogenic variants or VUS detected (Fig. 1). Proportions of pathogenic variants, pathogenic/VUS and VUS diagnosed between the two groups did not differ significantly (FET, *p* *=* 0.1848). A marginally higher proportion of subjects selecting a MCP had a pathogenic variant detected, either in isolation (9.7 vs. 8.2%) or in combination with a VUS (6.9 vs. 2.0%). Additionally, a MCP resulted in a VUS outcome more frequently than a BCP (32.9 vs. 22.4%), and patients opting for a BCP more frequently tested negative (67.3 vs. 50.5%) than those selecting a MCP. Almost 90.0% of pathogenic variants identified in those who selected a MCP would have been detected using a BCP (Table 2). Monoallelic pathogenic variants in genes associated with autosomal recessive conditions, including *MUTYH*-associated polyposis (familial adenomatous polyposis-2, FAP2; MIM: 608456) and Fanconi anaemia, complementation group I (FA; MIM: 609053), accounted for the remaining 10.0%.

**Table 2:** Spectrum of pathogenic variants detected in subjects who selected a MCP

Gene	MIM#	Number of pathogenic variants (*N* = 37)^a^		Detectable using a BCP
		*n*	%	
*BRCA1*	113705	14	37.8	Yes
*BRCA2*	600185	13	35.1	Yes
*ATM*	607585	2	5.4	Yes
*NBN*	602667	1	2.7	Yes
*PALB2*	610355	1	2.7	Yes
*PTEN*	601728	1	2.7	Yes
*TP53*	191170	1	2.7	Yes
*FANCI* ^b^	611360	1	2.7	No
*MUTYH* ^b^	604933	3	8.1	No

^a^One subject carried a pathogenic variant in both *BRCA2* and *MUTYH*

^b^Monoallelic state.

## Discussion

The NCCS CGS has employed a SDM approach for NGS panel testing since its inception in 2014, and the present study provides an overview of the selection patterns among breast and ovarian cancer patients. The vast majority of patients who proceed with NGS panel testing opt for broader NGS panels, often beyond the clinical indication. The majority of this cohort was referred for *BRCA1* (MIM: 113705) and *BRCA2* (MIM: 600185) testing, where a BCP would be sufficient on the basis of clinical indication and utility. This suggests that patients are willing to take on additional uncertainty in order to test larger numbers of genes, or perhaps place little significance on the relative levels of uncertainty between the offered panels. Previous studies of various stakeholder views on the return of unexpected findings from NGS research have demonstrated that genetic clinicians tend to hold more conservative views than the general public.^28,29^ Genetics clinicians typically express a preference for limiting the amount of information communicated in order to avoid unnecessarily causing anxiety or psychological distress to the patient. Additionally, there is concern regarding the testing of genes with limited clinical utility, where cancer risk estimates and medical management guidelines are not well established. Individuals from the general public were more concerned with ethical principles such as the right to autonomy and that ‘patients accept the consequences of any potential anxiety and uncertainty’.^29,30^ Our findings indicate that, in practice, patients do indeed accept a higher likelihood of uncertainty to examine a larger number of genes. This disconnect between the views of genetics clinicians, patients and the public should be carefully considered in the development of guidelines for the use of NGS panels.

Women who selected a MCP were significantly more likely to be diagnosed with ovarian cancer compared to those who selected a BCP. Broader MCPs contained genes with reduced penetrance for ovarian cancer risk compared with *BRCA1* and *BRCA2*, including the mismatch repair (MMR) genes (MIM: 120436, 609309, 600678, 600259) associated with Lynch syndrome (hereditary non-polyposis colorectal cancer, HNPCC [MIM: 609310, 120435, 614350, 614337]) and other genes involved in the homologous recombination pathway. Recent adjustments to the NCCN genetic testing criteria for Lynch syndrome (version 3.2017) now include an indication for ovarian cancer patients with a 5% or greater likelihood of detecting a pathogenic variant in a MMR gene calculated by certain prediction models. Previous versions used during the duration of our study suggested this as a consideration only. The frequency of pathogenic variants in the MMR genes is ~1% in ovarian cancer patients,^31–33^ and some authors have suggested this is sufficiently significant to warrant routinely offering testing of these genes.^31,32^ While clinical testing criteria is lacking for other moderate- and low-penetrance ovarian cancer genes, previous studies have shown that pathogenic variants are detected in ~3–5% of ovarian cancer cases (most commonly *BRIP1*; MIM: 605882).^32,33^ However, there is currently limited evidence regarding medical management for those who carry pathogenic variants in these moderate- and low- penetrance genes, although some have suggested recommending risk-reducing bilateral salpingo-oophorectomy (RRBSO) when the cumulative lifetime risk approaches or exceeds the lifetime risk of a woman with a first-degree relative affected with ovarian cancer who is *BRCA*-negative.^34^ Although the NCCN guidelines (version 1.2018) have emphasized the uncertainty regarding cancer risk estimates and medical management for reduced penetrance genes, they state that RRBSO can be considered from age 45–50 in individuals with pathogenic variants in *BRIP1, RAD51C*, and *RAD51D*. Our results suggest that despite the prospect of considerable uncertainty, SDM between ovarian cancer patients and clinicians leads to the testing of genes associated with ovarian cancer beyond *BRCA1* and *BRCA2*. It is interesting to note that for the ovarian cancer patients included in this study, no pathogenic variants were identified in these additional genes associated with ovarian cancer.

Conversely, patients who selected a BCP were more likely to have a personal history of breast cancer and be under age 50. Patients diagnosed with young-onset breast cancer face a plethora of unique challenges, including treatment-focused genetic testing, fertility preservation, and psychosocial issues^35^, and these factors may influence their level of uncertainty tolerance at the time of testing. Approximately one-third of breast cancer patients selected a BCP with a fast turnaround time for treatment purposes. Patient preferences regarding treatment decisions are important to explore as part of the SDM process and will have an influence on NGS panel selection, while fast turnaround testing is restricted to BCPs only. Additionally, there were more women under age 50 who were diagnosed with breast cancer than ovarian cancer in our cohort, suggesting this may be confounding the association with age.

In addition to a personal history of ovarian cancer, ethnicity was found to be most predictive of NGS panel selection. Interestingly, Chinese were significantly more likely to select a MCP compared to a BCP, suggesting there may be a higher tolerance of uncertainty in this group. Previous studies have demonstrated that Chinese have a high tolerance for risk with respect to financial decisions.^36–39^ Hsee and Weber^37^ demonstrated that when presented with investment choices that included a sure payoff option and a probabilistic payoff option, Chinese were more likely than Americans to choose the riskier latter option. However, this association was only true for investment decisions and not those of a health or academic nature. Our results suggest that there may be certain scenarios in health-related decision-making, where Chinese demonstrate similar patterns of tolerance for risk and uncertainty. Family decision-making, language barriers, and financial status have all been demonstrated to influence decision-making in Chinese American women regarding breast cancer treatment.^40,41^ Further research is needed to elucidate any underlying cultural factors contributing to these differences.

A surprising finding from this study is that various permutations of cancer family history were not found to be associated with test selection. Although family history is a common risk assessment tool for genetics clinicians to guide the use of genetic testing, patients may not place as much importance on family history for their decision-making. It may be that determining a possible hereditary cause for one’s own diagnosis is the predominant motivating factor for patients undergoing genetic testing in Singapore, and patients are therefore influenced less by family history. This is an important aspect to consider in pre-test counseling and highlights the need for discussion regarding how family history assists with genetic risk assessments.

Given the higher likelihood of VUS results with NGS panel testing, there has been great emphasis placed on limiting access to broader NGS panels unless necessary,^3–5,7,11^ particularly in understudied ethnic groups.^12^ Although our cohort demonstrated higher proportions of VUS results in the MCP group compared with the BCP, the difference was not statistically significant. This may mean that caution for using expanded panels based on the increased likelihood of VUS results could possibly be overemphasized, although this finding should be interpreted with caution as significance may be reached with a larger sample size. Nevertheless, the large number of VUS results echoes similar findings from a previous study of Singaporean breast cancer patients,^12^ emphasizing their recommendation that NGS panel testing be offered to patients only in the setting of a formal clinical cancer genetics service. It is also important to note that the vast majority of pathogenic variants identified in those who selected a MCP would have been detected with a BCP. While this may be interpreted as an indication for little benefit associated with broader testing, it would be important for in-depth exploration to understand the benefits from the patient perspective.

This study has strengths in the reliable and unbiased nature of the data presented, and the findings can be generalized to breast and ovarian cancer patients. However, there may be differences among patients with other cancer types, and so the results may not be representative of the entire population. This approach was employed in a center where thorough pre-test counseling is provided to patients by genetics clinicians, and so it is important to stress that the application of this approach may be inappropriate in the context of genetic testing ordered by non-genetics clinicians. This is an important consideration, particularly with mainstreaming implementation of genetic testing.^42,43^ Additionally, there is a wide range in the number of genes included in NGS panels defined as ‘MCPs’ and there may be variability within the groups that is unaccounted for. Finally, this study is limited by only analyzing recorded demographic and clinical factors on the CGS database; other possible influential factors such as education level and socio-economic status were not investigated.

The study employed a descriptive design and the data illustrates the overall patterns of NGS panel selection in relation to demographic and clinical variables using a SDM approach. Future studies employing both qualitative and quantitative methods are necessary to understand how patients cope with the uncertainty resulting from panel testing in both the short- and long-term, as well as explore the many aspects that may contribute to good quality decision-making.^26,44^ Interventions to standardize the way SDM is delivered in order to enhance the quality of decision-making should be considered such as the development of decision aids^44^ and appropriate clinician training.

We believe this is the first study evaluating factors associated with NGS panel selection using a SDM model of care where patients play an active role in the decision-making process. Our results indicate that the vast majority of patients opt for broader panels and it is personal factors, rather than family history factors, that are better predictors of this outcome. The SDM model along with evidence-based medicine are critical for high-quality health care.^45,46^ This approach aims to enhance patient empowerment by increasing the patient’s capacity to assess the available information critically and make informed health-related decisions autonomously.^47^ It is possible that SDM may balance the patient’s desire for autonomy in making decisions regarding their health care with the concerns of the genetic clinician. In assessing success of the SDM approach, further research is needed to evaluate patient outcomes and interventions to enhance patient decision-making. These results provide the necessary foundations for further research: to understand and evaluate the impact and subsequent outcomes when SDM is applied to NGS panel selection.

## Methods

### Subjects

Female patients with a personal history of breast and/or ovarian cancer who were referred to the CGS for genetic counseling were identified as potential subjects for inclusion. Patients were excluded from the study if they: (i) were under age 21 at the time of referral, (ii) had a previous diagnosis of a cancer other than breast and/or ovarian cancer, (iii) declined NGS panel testing, or (iv) were part of a family with a known familial pathogenic variant. These criteria were formulated to limit the number of potential confounding variables. Included subjects were not blood relatives. Written informed consent was taken at the point of genetic testing, and data was collected for subjects receiving their genetic test results in the period January 2014 to May 2017. The study was approved by the SingHealth Centralised Institutional Review Board (CIRB number is 2011/826/B).

### Shared decision-making model of care

**Fig. 2:**
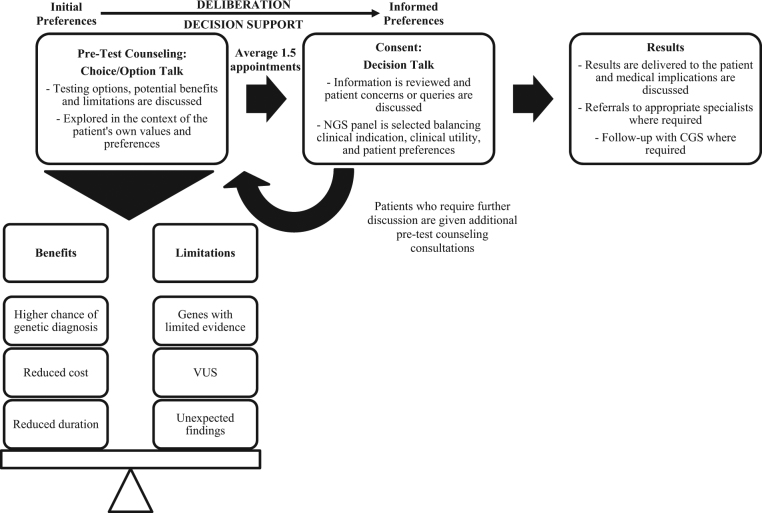
The National Cancer Centre Singapore model of care for genetic counseling, employing a shared decision-making (SDM) approach for NGS panel selection. It involves a process of the following three phases of communication: (i) choice talk, where the patient is made aware that choice exists; (ii) option talk, where patients are informed about the NGS panel options in greater detail; and (iii) decision talk, where patient decision-making and preferences are supported by exploring ‘what matters most to them’. Patients journey from their initial preferences to informed preferences, through a process of deliberation and along the way are provided with decision support by their clinician. VUS variant of uncertain significance, NGS next-generation sequencing, CGS Cancer Genetics Service

The NCCS CGS has adopted a SDM model of care for NGS panel selection (Fig. 2) and was guided by a SDM model proposed by Elwyn, et al.^48^ It involves a process of the following three phases of communication: (i) choice talk, where the patient is made aware that choice exists; (ii) option talk, where patients are informed about the NGS panel options in greater detail; and (iii) decision talk, where patient decision-making and preferences are supported by exploring ‘what matters most to them’. Patients journey from their initial preferences to informed preferences, through a process of deliberation. Methods to standardize the delivery of this model, such as decision aids, are yet to be developed.

Genetic counseling is provided to patients by a clinical cancer geneticist and/or genetic counselor (GC) prior to proceeding with genetic testing. During the study time period, the CGS was staffed by one clinical cancer geneticist and four GCs. In order to ensure consistency in the SDM approach, new recruits shadowed those with more experience in practicing under this model and were supervised until competent. During the initial consultation, medical and family history information was recorded, and the patient was educated regarding the benefits, implications and limitations of testing. This involves a thorough discussion regarding the following: (i) the suspected hereditary cancer syndrome(s) based on the medical/family history and whether genetic testing is clinically indicated, (ii) the types of results that can be reported and their respective implications, and (iii) the NGS panels available for testing.

All NGS panels that contained *at least BRCA1* and *BRCA2* were offered to all subjects and discussed in the context of their diagnosis and family history. The clinical indication for testing was determined using testing criteria (NCCN guidelines) or prediction models (Manchester scoring or BOADICEA), where a threshold of 10% or greater for the likelihood of detecting a pathogenic variant was met. Recent adjustments to the NCCN genetic testing criteria for Lynch syndrome (version 3.2017) now include an indication for ovarian cancer patients with a 5% or greater likelihood of detecting a pathogenic variant in a MMR gene calculated by certain prediction models. During the study time period, previous versions suggested this as a consideration only and subjects were counseled regarding the likelihood of a pathogenic variant, the potential benefits and limitations. Broader panels including genes with an associated risk of epithelial ovarian cancer, including MMR and moderate penetrance genes (e.g. *BRIP1, RAD51C, RAD51D)*, were discussed with subjects in the context of their diagnosis and the available evidence regarding cancer risks and medical management. In cases where subjects indicated a preference for limited testing and lower tolerance for uncertainty, the smallest NGS panel including *BRCA1* and *BRCA2* was selected. Subjects were given the option to test *BRCA1/2* alone, however, this was not selected by any included in this study.

Discussion regarding panel selection explores the careful balance between the information the subject is seeking, and the risks and sources of uncertainty. It is important that uncertainty be acknowledged in pre-test counseling and be explored with the patient. Through the processes of option and decision talk, their preferences and desires were elucidated through open dialogue between clinician and patient. Subjects were counseled regarding the sources of uncertainty: (i) the increased likelihood of detecting a VUS with every additional gene tested, (ii) the possibility of detecting a pathogenic variant in a gene with limited evidence regarding cancer risk estimates and medical management, and (iii) the possibility of detecting a pathogenic variant in a gene where there was no personal or family history of the associated phenotype (i.e., an unexpected finding), and the uncertainty of whether medical management should be altered. Subjects were encouraged to consider how they typically respond to uncertainty in situations that may be more relatable and to explore these scenarios in the context of their own values and preferences. For example, an analogy suitable for breast and/or ovarian cancer patients would be asking them how they would respond to an uncertain finding on a mammogram, where they would need further follow-up over time. Counseling techniques to check patient reactions and understanding, such as clarification and open-ended questioning, were used throughout. Supporting patients through this deliberation process aims to enable them to balance their preference for testing more (or fewer) genes with their general approach and tolerance of uncertainty.

Initial consultation duration times do vary and average 54.3 min (95% CI, 46.8–61.8). Patients were actively encouraged to take time to consider the information discussed. They were offered to return for a second consultation 1–2 weeks later to discuss their decision (unless otherwise requested by the patient) and genetic testing was arranged after obtaining informed consent. This interval provides patients with the option of additional time to consider the information presented and ensures queries are answered. Undecided patients were offered additional consultations if required. Patients opting for treatment-focused genetic testing usually proceed with testing at the first consultation with an urgent turnaround-time requested.

Testing is performed by clinical diagnostic laboratories in the USA certified under the Clinical Laboratory Improvement Amendments (CLIA). The cost of testing is the same for each NGS panel irrespective of the number of genes included. Patients who undergo genetic testing pay out of pocket, although subsidies are provided to certain patients based on financial need.^49^

### Data collection

Demographic and clinical data were gathered via the CGS database (REDCap Software, Version 6.10.3, 2017, Vanderbilt University). Demographic data included age, ethnicity and parity. Clinical data included personal cancer history, NGS panel type, genetic test result and family history (first-, second-, and/or third-degree relatives). The NGS panel type was categorized into the following two groups: (i) BCPs that included *BRCA1/2*, as well as other predominantly high- and occasionally moderate-penetrance breast cancer genes (range from 7 to 11 genes; Supplementary Table 1), and (ii) MCPs that include genes with associated risks of other cancers in addition to those included in the BCPs (range from 19 to 80 genes; Supplementary Table 2). Specific information regarding the classification of NGS panels and tested genes are delineated in the Supplementary Material (Supplementary Tables 1–3). Testing with a fast turnaround time for treatment purposes could only be accessed when selecting a BCP.

### Data analysis

Comparisons of demographic variables and clinical characteristics (all categorical) between subjects who selected BCPs and MCPs were performed using a two-sided Fisher’s exact test. Variables significant at *p* < 0.05 were included as candidates in a stepwise multiple logistic regression analysis (significance levels to enter and stay of 0.20 and 0.25, respectively) for the purpose of identifying a parsimonious subset of independent predictors of panel type. Accuracy of the predictive model was assessed via area under the ROC curve (AUC). All statistical analyses were performed using SAS software version 9.3 (2011).

### Data availability

All the data generated or analyzed during this study are included in this published article (and the supplementary material).

## Electronic supplementary material

Supplementary Material
