# Supplementary material for: Predictors of next-generation sequencing panel selection using a shared decision-making approach
Source: NPJ Genom Med. 2018 Apr 27;3:11. doi: 10.1038/s41525-018-0050-y (PMC5923203; doi:10.1038/s41525-018-0050-y)
Supplement: Supplementary file 1 — Supplementary Material [file 41525_2018_50_MOESM1_ESM.pdf]

SUPPLEMENTARY TABLES

Supplementary Table 1. NGS panels classified as BCPs

Laboratory 1			Laboratory 2	
BCP 1	BCP 2	BCP 3	BCP 4	BCP 5
BRCA1	*ATM*	*ATM*	*ATM*	*ATM*
BRCA2	*BRCA1*	*BRCA1*	*BRCA1*	*BRCA1*
CDH1	*BRCA2*	*BRCA2*	*BRCA2*	*BRCA2*
PALB2	*CDH1*	*CDH1*	*CDH1*	*CDH1*
PTEN	*CHEK2*	*CHEK2*	*CHEK2*	*CHEK2*
STK11	*PALB2*	*NBN*	*PALB2*	*NBN*
TP53	*PTEN*	*NF1*	*PTEN*	*PALB2*
	STK11	*PALB2*	*TP53*	*PTEN*
	TP53	*PTEN*		*TP53*
		STK11		
		TP53		

Abbreviation: BCP, breast cancer panel

Supplementary Table 2. NGS panels classified as MCPs

Laboratory 1					Laboratory 2			Laboratory 3
MCP 1	MCP 2	MCP 3	MCP 4	MCP 5[1] (Add-on FA)	MCP 6	MCP 7	MCP 8	MCP 9
ATM	*ATM*	*APC*	*ALK*	*BRCA2*	*ATM*	*APC*	*APC*	*ATM*
BRCA1	*BARD1*	*ATM*	*APC*	*BRIP1*	*BARD1*	*ATM*	*ATM*	*BRCA1*
BRCA2	*BRCA1*	*AXIN2*	*ATM*	*ERCC4*	*BRCA1*	*BMPR1A*	*AXIN2*	*BRCA2*
BRIP1	*BRCA2*	*BARD1*	*AXIN2*	*FANCA*	*BRCA2*	*BRCA1*	*BARD1*	*BRIP1*
CDH1	*BRIP1*	*BMPR1A*	*BAP1*	*FANCB*	*BRIP1*	*BRCA2*	*BMPR1A*	*CDH1*
CHEK2	*CDH1*	*BRCA1*	*BARD1*	*FANCC*	*CDH1*	*BRIP1*	*BRCA1*	*CHEK2*
EPCAM	*CHEK2*	*BRCA2*	*BLM*	*FANCD2*	*CHEK2*	*CDH1*	*BRCA2*	*EPCAM*
MLH1	*EPCAM*	*BRIP1*	*BMPR1A*	*FANCE*	*EPCAM*	*CDKN2A*	*BRIP1*	*MLH1*
MSH2	*FANCC*	*CDH1*	*BRCA1*	*FANCF*	*FANCC*	*CHEK2*	*CDH1*	*MSH2*
MSH6	*MLH1*	*CDKN2A*	*BRCA2*	*FANCG*	*MLH1*	*EPCAM*	*CDK4*	*MSH6*
NBN	*MRE11A*	*CHEK2*	*BRIP1*	*FANCI*	*MSH2*	*MLH1*	*CDKN2A*	*NBN*
NF1	*MSH2*	*DICER1*	*CASR*	*FANCL*	*MSH6*	*MSH2*	*CHEK2*	*NF1*
PALB2	*MSH6*	*EPCAM*	*CDC73*	*FANCM*	*NBN*	*MSH6*	*EPCAM*	*PALB2*
PMS2	*NBN*	*GREM1*	*CDH1*	*PALB2*	*PALB2*	*MUTYH*	*FANCC*	*PMS2*
PTEN	*NF1*	*KIT*	*CDK4*	*RAD51C*	*PMS2*	*PALB2*	*GREM1*	*PTEN*
RAD51C	*PALB2*	*MEN1*	*CDKN1B*	*SLX4*	*PTEN*	*PMS2*	*MLH1*	*RAD51C*
RAD51D	*PMS2*	*MLH1*	*CDKN1C*	*XRCC2*	*RAD51C*	*PTEN*	*MSH2*	*RAD51D*
STK11	*PTEN*	*MSH2*	*CDKN2A*		*RAD51D*	*RAD51C*	*MSH6*	*STK11*
TP53	*RAD50*	*MSH6*	*CEBPA*		*TP53*	*RAD51D*	*MUTYH*	*TP53*
	RAD51C	*MUTYH*	*CHEK2*		*XRCC2*	*SMAD4*	*NBN*	
	RAD51D	*NBN*	*DICER1*			*STK11*	*PALB2*	
	STK11	*NF1*	*DIS3L2*			*TP53*	*PMS2*	
	TP53	*PALB2*	*EGFR*			*VHL*	*POLD1*	
		PDGFRA	*EPCAM*				*POLE*	
		PMS2	*FH*				*PTEN*	
		POLD1	*FLCN*				*RAD51C*	
		POLE	*GATA2*				*RAD51D*	

	PTEN<br>*RAD50*<br>*RAD51C*<br>*RAD51D*<br>*SDHA*<br>*SDHB*<br>*SDHC*<br>*SDHD*<br>*SMAD4*<br>*SMARCA4*<br>*STK11*<br>*TP53*<br>*TSC1*<br>*TSC2*<br>*VHL*	*GPC3*<br>*GREM1*<br>*HOXB13*<br>*HRAS*<br>*KIT*<br>*MAX*<br>*MEN1*<br>*MET*<br>*MITF*<br>*MLH1*<br>*MSH2*<br>*MSH6*<br>*MUTYH*<br>*NBN*<br>*NF1*<br>*NF2*<br>*PALB2*<br>*PDGFRA*<br>*PHOX2B*<br>*PMS2*<br>*POLD1*<br>*POLE*<br>*POT1*<br>*PRKAR1A*<br>*PTCH1*<br>*PTEN*<br>*RAD50*<br>*RAD51C*<br>*RAD51D*<br>*RB1*<br>*RECQL4*<br>*RET*	*SMAD4*<br>*STK11*<br>*TP53*<br>*VHL*<br>*XRCC2*	

RUNX1<br>*SDHA*<br>*SDHAF2*<br>*SDHB*<br>*SDHC*<br>*SDHD*<br>*SMAD4*<br>*SMARCA4*<br>*SMARCB1*<br>*SMARCE1*<br>*STK11*<br>*SUFU*<br>*TERC*<br>*TERT*<br>*TMEM127*<br>*TP53*<br>*TSC1*<br>*TSC2*<br>*VHL*<br>*WRN*<br>*WT1*		

[1]MCP 5 is an 'add-on' panel that is selected in addition to MCP 1-4.
Abbreviation: MCP, multi-cancer panel.

Supplementary Table 3. NGS panel selection when divided into three categories, per personal cancer history

	Breast cancer only		Ovarian cancer only		Breast and ovarian cancer	
	n	%	n	%	n	%
NGS panel						
Breast cancer panel[1]	45	26.0	2	2.8	2	10.0
Br/Gyn cancer panel[2]	75	43.4	31	43.0	9	45.0
Multi-cancer panel[3]	53	30.6	39	54.2	9	45.0
Total	173	100.0	72	100.0	20	100.0

[1] All panels classified as BCP (see Supp. Table 1)
[2] MCP1, MCP2, MCP6, MCP9 (see Supp. Table 2)
[3] MCP3, MCP4, MCP7, MCP8 (see Supp. Table 2)
Abbreviation: Br/Gyn, breast and gynaecological cancer panel.
